# A multivariate relationship between the kinematic and clinical parameters of knee osteoarthritis population

**DOI:** 10.1186/s12938-019-0676-8

**Published:** 2019-05-15

**Authors:** Fatima Bensalma, Neila Mezghani, Youssef Ouakrim, Alexandre Fuentes, Manon Choinière, Nathalie J. Bureau, Madelaine Durand, Nicola Hagemeister

**Affiliations:** 1 Centre de Recherche LICEF, TELUQ, 5800, rue Saint-Denis, bureau 1105, Montreal, Canada; 2 Laboratoire de recherche en Imagerie et Orthopédie (LIO), Research Center of the Centre hospitalier de l’Université de Montréal (CRCHUM), 900, rue Saint-Denis, Montreal, Canada; 3 Research Center of the Centre hospitalier de l’Université de Montréal (CRCHUM), 900, rue Saint-Denis, Montreal, Canada; 4 École de Technologie Superieure, 1100, rue Notre-Dame Ouest, Montreal, Canada; 5 Department of Anesthesiology University and Pain Medicine, Faculty of Medicine, Université de Montréal, 2900, boul. Édouard-Montpetit, Montreal, Canada; 6 Department of Radiology, Faculty of Medicine, Université de Montréal, 2900, boul. Édouard-Montpetit, Montreal, Canada; 7 Department of Medicine, Université de Montréal, 2900, boul. Édouard-Montpetit, Montreal, Canada

**Keywords:** Biomechanics, Canonical correlation, Multivariate analysis, Multiple regression, Kinematic gait analysis, Knee osteoarthritis (OA)

## Abstract

**Background:**

Biomechanical and clinical parameters contribute very closely to functional evaluations of the knee joint. To better understand knee osteoarthritis joint function, the association between a set of knee biomechanical data and a set of clinical parameters of an osteoarthritis population (OA) is investigated in this study.

**Methods:**

The biomechanical data used here are a set of characteristics derived from 3D knee kinematic patterns: flexion/extension, abduction/adduction, and tibial internal/external rotation measurements, all determined during gait recording. The clinical parameters include a KOOS questionnaire and the patient’s demographic characteristics. Canonical correlation analysis (CCA) is used (1) to evaluate the multivariate relationship between biomechanical data and clinical parameter sets, and (2) to cluster the most correlated parameters. Multivariate models were created within the identified clusters to determine the effect of each parameter’s subset on the other. The analyses were performed on a large database containing 166 OA patients.

**Results:**

The CCA results showed meaningful correlations that gave rise to three different clusters. Multivariate linear models were found explaining the subjective clinical parameters by evaluating the biomechanical data contained within each cluster.

**Conclusion:**

The results showed that a multivariate analysis of the clinical symptoms and the biomechanical characteristics of knee joint function allowed a better understanding of their relationships.

## Background

Biomechanical knee assessment is increasingly used in gait analysis as a tool for characterizing the knee function [1], understanding pathological knee alterations [2], and assessing the progression of knee pathologies and their impact on gait [3]. It has already been suggested that the type and severity of biomechanical changes should be assessed since they can impact treatment outcomes [4]. Mechanical factors linked to the progression of osteoarthritis (OA) [5] and its treatment [6] have also been identified. Still, the relationship between the kinematic and clinical parameters of knee OA populations has not been sufficiently explained and remains incompletely understood. Only a few studies have investigated the relationship between 3D knee kinematic parameters and clinical data [7, 8]. These studies have been limited to a univariate analysis implying the correlation between one kinematic parameter and one specific clinical parameter. Such analysis is not adapted to the complexity of biomechanical data [9] and can even mask several strong relationships if the parameters are considered independently.

The objective of this study is (1) to evaluate the multivariate relationship (compared to the univariate approach) between a set of biomechanical data and a set of clinical parameters of an osteoarthritis population, and (2) to cluster the most correlated parameter. The biomechanical data are a set of characteristics extracted from 3D knee kinematic patterns during gait recording: flexion/extension, abduction/adduction, and tibial internal/external rotation measurements. The clinical parameters were acquired via the Knee Osteoarthritis Outcome Score (KOOS) questionnaire. Through this questionnaire, the patient provides a valid and reliable assessment of his/her health status relative to the pathology [10]. Our hypothesis is that these subjective clinical measures may complement objective biomechanical measures for a better understanding of knee joint function.

This study utilizes a canonical correlation analysis (CCA) to evaluate the relationship between a set of biomechanical data and a set of clinical parameters of an osteoarthritis population. CCA is a method for exploring the relationship between two multivariate sets of variables all measured on the same individual. Although the CCA has already been successfully applied to several applications in image processing [11] and in the domain of ecology [12], its use remains almost limited in the biomedical field. This situation could be due to the difficulty of interpreting results. To our knowledge, this study is the first to consider such a multivariate analysis combined with multivariate modeling in the biomechanical domain.

## Methods

**Fig. 1:**
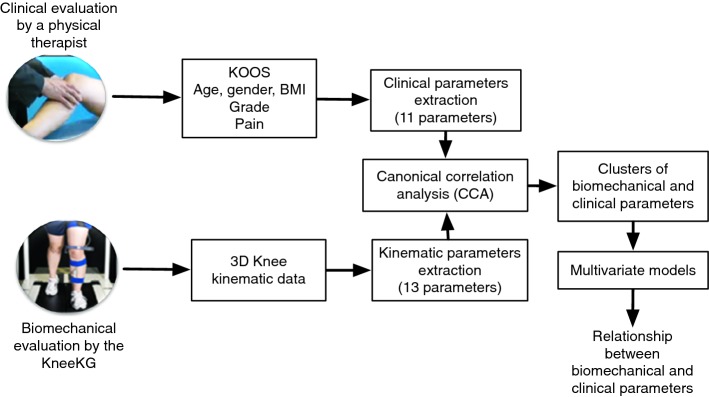
Steps of the proposed method

The flowchart of the proposed method is shown in Fig. 1. The first step consists of biomechanical and clinical data acquisition and parameter extraction. Next is a multivariate analysis using a CCA, which aims at clustering the most correlated parameters. Multivariate models are then developed within the identified clusters to determine the correlation and relationships between biomechanical and clinical data.

### Biomechanical and clinical data collection

**Fig. 2:**
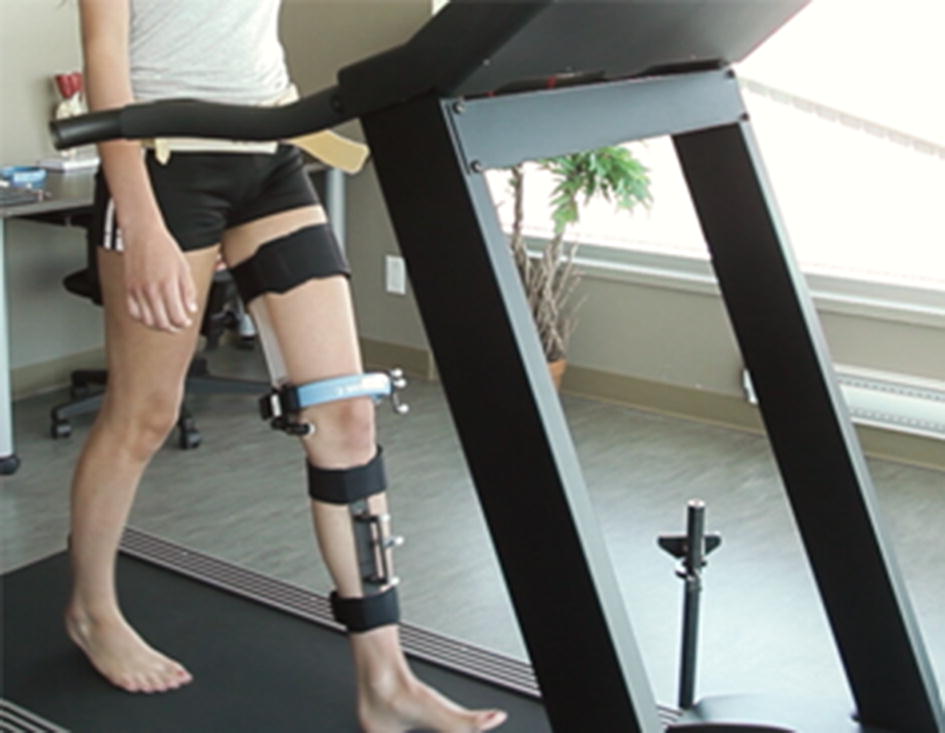
KneeKG acquisition system

One hundred and sixty-six patients with clinically and radiographically confirmed knee osteoarthritis participated in the study [mean age of 62 years old ($\text {SD}=9.2$), body mass index (BMI) of 32 kg/m^2^ ($\text {SD}=7.3$), 99 women ($59.6\%$)]. The experimental data were collected by the KneeKG (Emovi, Canada), a knee marker attachment system (Fig. 2) designed to reduce skin-motion artifacts during motion [13]. The KneeKG was installed on participants knees to record 3D kinematics (flexion/extension, abduction/adduction, and internal/external rotation) during gait trials. The kinematic data were represented over several gait cycles (GCs) and averaged to obtain mean GCs per participant. This was followed by re-sampling of from 1 to 100% of the GCs with 100 measurement points for each participant in each plane.

Participants were also asked to answer the KOOS questionnaires. The KOOS is a valid and reliable instrument which assesses the impact of knee OA on five domains: symptoms, pain, activities of daily living (ADL), sports and recreation (Sports/Rec), and quality of life (QoL). Scores on the subscales range from 0 (extreme symptoms) to 100 (no symptoms) [10].

### Biomechanical and clinical parameters’ extraction

For each participant, a set of 69 parameters that correspond to biomechanical values were measured on the kinematics curves from gait analysis. These parameters were chosen based on variables routinely assessed in clinical biomechanical studies of knee OA populations, such as maximums, minimums, varus and valgus thrust, angles at initial contact, mean values, and range of motion (ROM) throughout GCs or GC sub-phases (i.e., loading, stance, swing) [14]. Thirteen parameters among this set have been identified by Mezghani et al. [3] as having the potential to serve as diagnostic and burden of disease biomarkers of knee OA. The kinematic parameters considered as biomarkers were identified by incremental selection on a regression tree determining the best set of biomechanical parameters for each biomarker type: knee OA disease diagnosis and severity grading. This has been done in accordance with the standard BIPED (burden of disease, investigative, prognostic, efficacy of intervention, and diagnostic) OA biomarker classification scheme [15]. Table 1 describes the clinical meaning of the 13 biomechanical data considered in this study.

**Table 1:** Description of the 13 biomechanical parameters

**Y**	Name	Description	Mean (SD)
$Y_{1}$	Rot_RomSw	Range of motion of internal/external rotation during swing	1.9 (1.2)
$Y_{2}$	Abd_MeanSt	Mean abduction/adduction angle during the stance phase	4.4 (5.7)
$Y_{3}$	Rot_Init	Internal/external rotation angle at initial contact	3.0 (3.6)
$Y_{4}$	Flex_Max	Flexion angle maximum	59.5 (5.7)
$Y_{5}$	Flex_EndSt	Flexion angle at the end of the stance phase	12.1 (7.2)
$Y_{6}$	Abd_MaxSw	Maximum of the abduction/adduction angle during swing phase	7.9 (5.6)
$Y_{7}$	Abd_MinLo	Minimum abduction/adduction angle during the loading phase	4.3 (5.5)
$Y_{8}$	Abd_Init	Abduction/adduction angle at initial contact	4.8 (5.3)
$Y_{9}$	Abd_Lo	Abduction/adduction angle at the end of the loading phase	5.6 (5.9)
$Y_{10}$	Abd_Rom	Range of motion of the abduction/adduction angle	9.3 (3.1)
$Y_{11}$	Rot_InitAbs	Internal/external rotation absolute angle value at initial contact	3.8 (2.8)
$Y_{12}$	Rot_Rom	Range of motion of the internal/external rotation	11.3 (3.2)
$Y_{13}$	Abd_RomLo	Range of motion of the abduction/adduction angle during loading phase	−1.3 (2.4)

The participants in this study were selected if the OA was the main cause of their knee pain. The exclusion criteria were considered for the subjects being on a waiting list for total knee replacement. Patients being pregnant, suffering from rheumatoid arthritis, and active cancer were also excluded. A standardized radiographic examination of both knees was performed after the patient had given written informed consent. Only patients who had a Kellgren–Lawrence (KL) grade ≥ 2 on radiographs were considered and only data from the most painful knee were collected.

The set of clinical parameters contains 11 measurements: the patients’ demographic characteristics (sex, age, BMI), the degree of osteoarthritis severity variable (grade), the variable (pain) which is measured on the Pain Numerical Scale (NS) for Knees (on which no pain is marked 0 and the worst pain imaginable is marked 10), and 6 scores generated using the KOOS questionnaire [10, 16]. These scores assess the five dimensions mentioned above. An overall KOOS score is then generated and normalized to give a maximum of 100 points in the absence of pain or other knee dysfunction. Through this questionnaire, the patient provides a valid and reliable assessment of his health status relative to the pathology [10, 16]. A summary of the clinical parameters and their descriptions is provided in Table 2.

**Table 2:** Description of the clinical parameters

**X**	Name	Description	Mean (SD)
$X_{1}$	Grade (1 to 4)	Degree of osteoarthritis severity	3.0 (0.8)
$X_{2}$	Pain (4 to 10)	Outcome score for pain	6.6 (1.8)
$X_{3}$	Sex (1: Men, 0: Women)	Gender	Men:* n* = 68; Women:* n* = 98
$X_{4}$	Age	Age (years)	61.9 (9.2)
$X_{5}$	BMI	Body mass index (kg/m^2^)	31.8 (7.3)
$X_{6}$	KOOS_Symptoms	KOOS score for symptoms	62.7 (17.4)
$X_{7}$	KOOS_Pain	KOOS score for pain	60.5 (17.3)
$X_{8}$	KOOS_Adl	KOOS score for daily living	67.4 (18.3)
$X_{9}$	KOOS_Sport	KOOS score for sport and recreation function	38.7 (25.7)
$X_{10}$	KOOS_Qol	KOOS score for quality of life	52.3 (22.8)
$X_{11}$	KOOS	Normalized overall KOOS score	56.3 (17.1)

### Canonical correlation analysis (CCA)

The CCA is a multivariate statistical technique that explores the correlations between two sets of variables observed on the same individual [17]. The theoretical development of CCA can be found in [18–20]. Let **Y** $= [Y_1, Y_2, ... , Y_q]$ and $\mathbf{X} = [X_1, X_2, ... , X_p]$ denote the two data vectors to be analyzed, i.e., the biomechanical parameter vector and the clinical parameter vector. In our case $q =13$ and $p=10$. The **Y**-variables can be thought of as response (or dependent) variables, but in fact, the **X** and the **Y** sets can be interchanged without affecting the results. The aim of the CCA is to project **X** and **Y** datasets onto basis vectors $\mathbf{A}$ and $\mathbf{B},$ respectively, in a way that the correlations between the projections of the variables onto these basis vectors are mutually maximized [21]:

1 $$\begin{aligned} \rho=   \max _{\mathbf{A}, \mathbf{B}} \; \text{corr}(\mathbf{X A}, \mathbf{Y B}), \end{aligned}$$

where $\rho$ is the Pearson correlation coefficient vector and $\mathbf{U}$ and $\mathbf{V}$ are linear combinations of the original variables $\mathbf{X}$ and $\mathbf{Y}$ (Eqs. 2, 3), respectively.

2 $$\begin{aligned} \mathbf{U}=  a_{1} X_1 + a_{2} X_2 + \cdots + a_{p} X_p = \mathbf{X A} \end{aligned}$$

3 $$\begin{aligned} \mathbf{V}= b_{1} Y_1 + b_{2} Y_2 + \cdots + b_{q} Y_q = \mathbf{Y B}. \end{aligned}$$

**Fig. 3:**
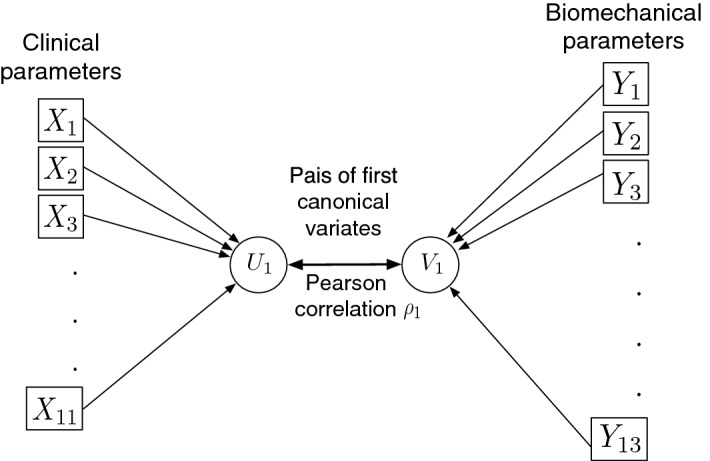
Path diagram of canonical correlation analysis

$\mathbf{U}$ and $\mathbf{V}$ are the canonical variate vectors. The coefficient vectors $\mathbf{A}$ and $\mathbf{B}$ are known as canonical weights, canonical vectors, or canonical coefficients. The procedure is to find the first two canonical variates $U_{1}$ and $V_{1}$ that have the largest correlation as illustrated in Fig. 3. The maximized correlation between these two canonical variates is the first canonical correlation $\rho _{1}$. The canonical coefficients are normalized such that each canonical variate has a variance of 1. The procedure continues by finding a second pair of canonical variates $U_{2}$ and $V_{2}$, uncorrelated with the first pair, that produces the second highest correlation coefficient $\rho _{2}$. The process continues until the number of pairs of canonical variables reaches pre-set min(*p*, *q*).

To evaluate the statistical significance of the canonical correlation model, we use the Wilks’ Lambda statistic ($\lambda$). This is a multivariate statistic that uses approximations based on the Fisher distribution for the null hypothesis, i.e., all canonical correlations are zero in the population. The small *p* values for this test $(< 0.05)$ suggest a rejection of the null hypothesis and that the first canonical correlation is significant. In our study, the analysis was conducted using the R software environment for Statistical Computing (R version 3.4.3) [22].

### Comparison between the multivariate analysis (CCA) and a univariate analysis

The results of the CCA analysis were compared to those of a univariate analysis based on the pairwise correlation matrix calculated using the Pearson correlation coefficient. The objective of this comparison is to show that the univariate analysis cannot adapt to the complexity of biomechanical data [9] and can even mask several strong relationships if parameters are considered individually.

### Clustering via correlation biplot

The results of a CCA are visualized by a correlation biplot graph, which represents the between-set correlation matrix $R_{\mathbf{X}{} \mathbf{Y}}$ by a joint plot. This format allows for the visualization of the intra-set correlation for the original variables and the corresponding canonical variates and of the correlation between the original variables and the opposite canonical variates. The main features of a correlation biplot are the angles between the variables from sets $\mathbf{X}$ and $\mathbf{Y}$ in the biplot, which reflect their correlations [12]. The combined angle and direction of the $\mathbf{X}$ and $\mathbf{Y}$ variables indicate the importance of the positive and negative correlations of the two sets. Strongly correlated variables are very close to each other. More specifically, in our case, the correlation biplot graph is used to cluster biomechanical data and clinical parameters. The identified clusters are then used to explain the relationships between the sets of parameters within the clusters.

### Canonical prediction model and regression within clusters

Once the clusters are identified, we can explain the relationship between the parameters within the clusters using a regression analysis. This analysis aims at estimating the coefficients of the linear equation, involving one or more independent variables (clinical parameters) that best predict the value of the dependent variables (biomechanical data). The purpose of regression is to predict $\mathbf{X}$ on the basis of $\mathbf{Y}$ within the clusters.

In order to determine which variable should be considered as dependent and which as independent, we performed a redundancy analysis. This analysis measures the proportion of variance of one original variable explained by the canonical variate of the other set. The original variables of one set are well represented by the canonical variate of the other set when the redundancy index is higher. A relational model is then proposed to determine which of the variables best explains the other. A redundancy coefficient close to 1 is considered to be the highest, and shows that the amount of the dependent (original) variable’s variance shared with the independent (canonical) variable is significant, and vice versa; a coefficient close to zero means that there is no significance in the shared variance.

## Results

### Univariate correlation analysis

**Fig. 4:**
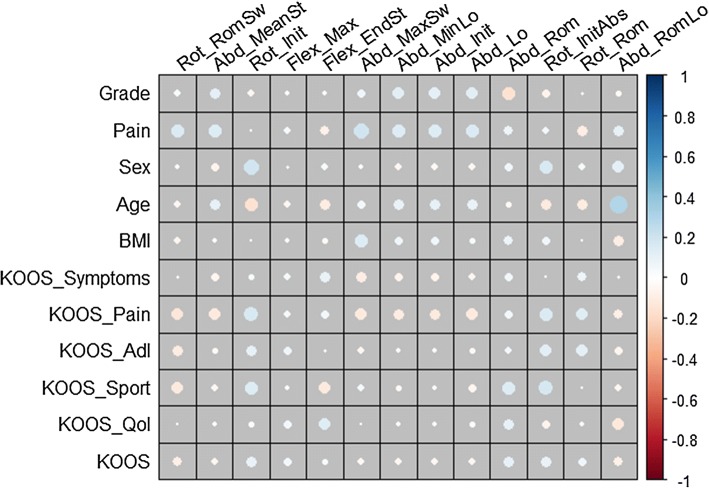
Visualization of the correlation matrix

The univariate correlation matrix is visualized in a graphical display in Fig. 4. The 10 clinical parameters are in rows and the 13 biomechanical parameters are in columns. Positive correlations are displayed in blue and negative correlations in red color. Color intensity and the size of the circle are proportional to the correlation coefficients. The correlations between the biomechanical data and clinical parameters are moderate. The largest correlation value is between age ($X_{4}$) and the range of motion of the abduction/adduction angle during loading phase ($Y_{13}$: Abd_RomLo) $(r=0.3)$. Indeed, the univariate analysis considers the pairwise correlation of only two parameters. This result supports the need for a multivariate investigation.

### Canonical correlations and multivariate statistic

The Wilks’ Lambda statistics of the canonical correlation model was $\lambda =0.32$,* p* = 0.04. This confirms that canonical correlations are worthy of consideration and the between-set correlations are significant. The two first higher canonical correlations are $\rho _{1}=0.52$ and $\rho _{2}=0.44$.

### Correlation clustering via biplot

**Fig. 5:**
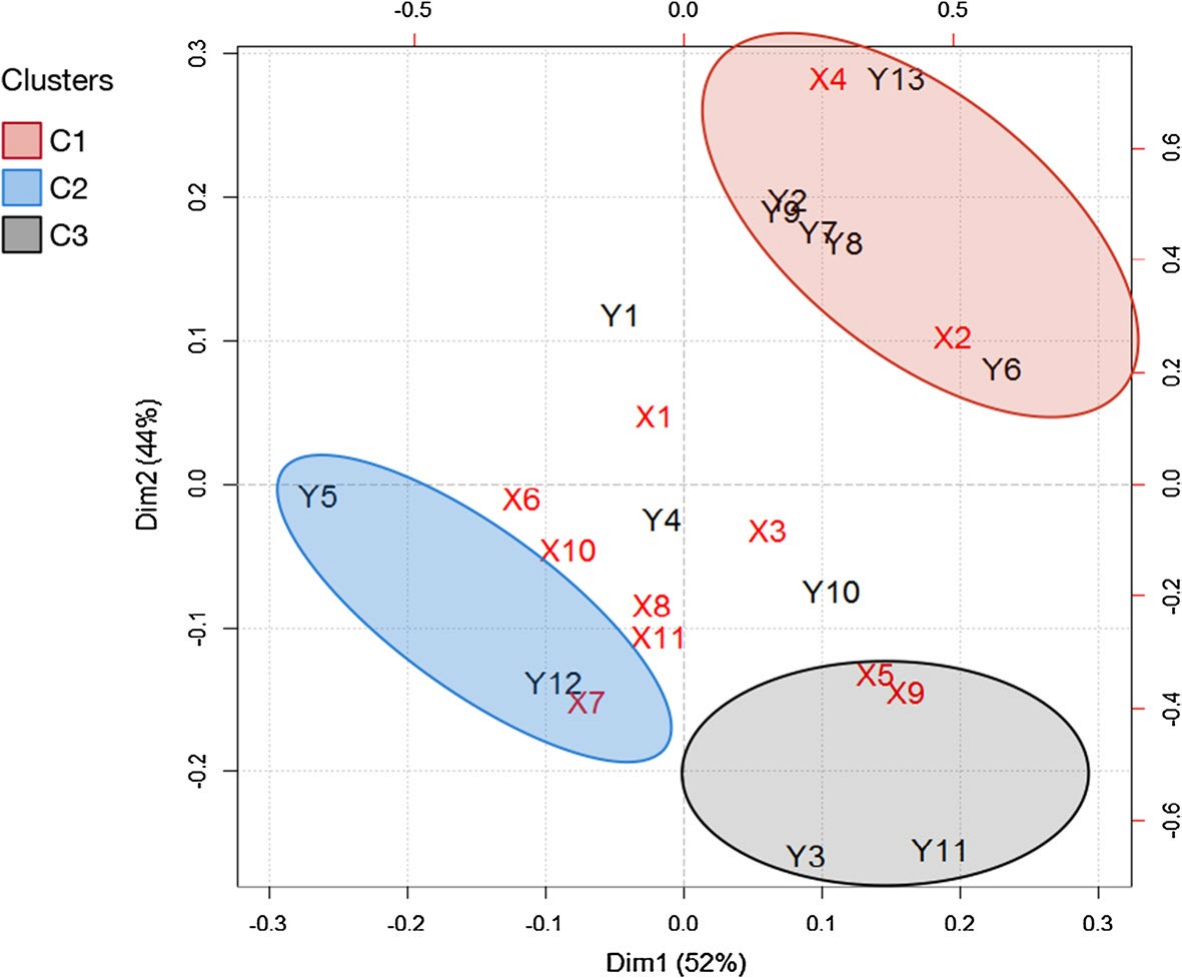
Biplot visualization of the between-set correlations. The three ovals correspond to the three clusters

The correlation biplot graph of Fig. 5 represents the between-set correlation matrix $R_{\mathbf{X}{} \mathbf{Y}}$, i.e., the correlation between 13 biomechanical parameters (in black) and 10 clinical parameters (in red) via their canonical variates. It identifies three clusters, each grouping biomechanical and clinical parameter. Recall that, the 13 biomechanical parameters among this set have been identified by Mezghani et al. [3] as having the potential to serve as diagnostic and burden of disease biomarkers of knee OA.

Note that the correlations located around the center are negligible. For example, the parameters $X_1$, $X_3$, $Y_4$ , and $Y_{10}$ are very close to the origin in the biplot, which shows that they are not important in the CCA. Meanwhile, the parameters $Y_2$, $Y_7$ , and $Y_9$ are highly correlated with $Y_8$. Thus, these parameters have been removed from the subsequent analysis, and the identified clusters between $\mathbf{X}$ and $\mathbf{Y}$ are summarized in Table 3.

**Table 3:** Description of the retained correlation clusters between $\mathbf{X}$ and $\mathbf{Y}$

Cluster	$\mathbf{X}$	$\longleftrightarrow$	$\mathbf{Y}$
$\mathbf{C_{1}}$	$X_{4}$, $X_{2}$	$\longleftrightarrow$	$Y_{6}$, $Y_{8}$, $Y_{13}$
$\mathbf{C_{2}}$	$X_{7}$	$\longleftrightarrow$	$Y_{5}$, $Y_{12}$
$\mathbf{C_{3}}$	$X_{5}$, $X_{9}$	$\longleftrightarrow$	$Y_{3}$, $Y_{11}$

### Canonical correlation model

**Fig. 6:**
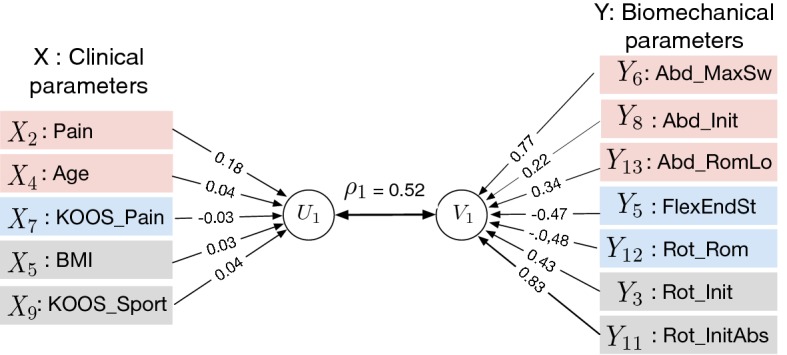
Canonical correlation model

The canonical model of the first canonical variates is summarized in Fig. 6. This model describes the most strongly correlated variables with their appropriate weights or canonical coefficients. It provides the following two relations with a significant canonical correlation relation ($\rho _{1} = 0.52, p=0.04$):

$$\begin{aligned} & V_{1} = {} 0.43~Y_3 -0.47~Y_5 + 0.77~Y_6 + 0.22~Y_8 + 0.83~Y_{11} -0.48~Y_{12} + 0.34~Y_{13} \\ & U_{1}=  {} 0.18~X_2 + 0.04~X_4 + 0.03~X_5 + 0.04~X_7 -0.03~X_9 \end{aligned}.$$

### Redundancy coefficients

The total redundancy corresponds to $8.98\%$ of the variance of $\mathbf{X}$ explained by the opposite canonical variate $\mathbf{V}$, and to $8.71\%$ of the variance of $\mathbf{Y}$ explained by the opposite canonical variate $\mathbf{U}$. We can therefore affirm the equality of the indices of shared variances; more specifically, both clinical and biomechanical parameters may be considered as dependent or independent.

### Regression within the clusters

Following the redundancy analysis, we developed a multivariate regression model within clusters to investigate the relationships between clinical and biomechanical parameters. Table 4 summarizes the regression model developed for each cluster. All the estimated regression models were significant with an Adjusted $R^{2} \ge 0.68$. The residual standard errors RSEs of the models were $0.63\le \text{RSE} \le 1.05$ indicating a perfect fit to the data by the estimated models.

**Table 4:** Multiple linear regression models

Cluster	Variables	Coefficient (Std. error)	*P* value	Residual Std. rrror	Adjusted $R^{2}$	*P* value of* F* statistic
$\mathbf{C_{1}}$	$X_{2}{:}\; \text{Pain~regression~model}$			0.68	0.70	< 0.01
	$Y_{6}$: Abd_MaxSw	0.19 (0.02)	< 0.01			
	$Y_{8}$: Abd_Init	− 0.33 (0.08)	< 0.01			
	$Y_{13}$: Abd_RomLo	− 0.14 (0.06)	0.035			
$\mathbf{C_{2}}$	$X_{7}{:}\; \text{KOOS\_Pain~regression~model}$			0.63	0.89	< 0.01
	$Y_{12}$: Rot_Rom	0.26 (0.02)	< 0.01			
	$Y_{5}$: Flex_EndSt	0.06 (0.01)	< 0.01			
$\mathbf{C_{3}}$	$X_{9}{:}\; \text{KOOS\_Sport~regression~model}$			1.05	0.68	< 0.01
	$Y_{3}$: Rot_Init	0.12 (0.05)	0.02			
	$X_{5}$: BMI	1.39 (0.11)	< 0.01			

## Discussion

### Cluster 1 analysis

The cluster $\mathbf{C}_{1}$ regroups biomechanical data corresponding to kinematic parameters in the frontal plane (abduction/adduction) ($Y_6$: Abd_MaxSw, $Y_8$: Abd_Init and $Y_{13}$: Abd_ROMLo) and the level of pain ($X_2$) as described in Tables 1, 2, and 3. The results of the multivariate regression of pain as a function of three parameters of the abduction/adduction movement (the $X_{2}$: Pain regression model in Table 4) indicate that the pain felt is negatively correlated with $Y_8$ and $Y_{13}$, while positively correlated with $Y_6$.

### Cluster 2 analysis

From the second cluster $\mathbf{C_{2}}$, the Flexion angle at the end of the stance phase ($Y_{5}$ :Flex_EndSt), the Range of motion of the internal/external rotation ($Y_{12}$ :Rot_Rom), and the pain measured by the score KOOS ($X_{7}$ :KOOS_Pain) were very directly related. The association between the improvement in KOOS_Pain score and changes in the range of motion (ROM) in the transverse plane was identified by Makovey et al. [23]. The subjective value of KOOS_pain is positively correlated with parameters in the sagittal (flexion/extension) and transverse (internal/external rotation) plane as shown by the $X_{7}$ :KOOS_Pain regression model in Table 4.

### Cluster 3 analysis

From the third cluster $\mathbf{C_{3}}$, only kinematic parameters in the transverse plane (internal/external rotation) $Y_{3}$ presented correlations with $X_{5}$ (BMI) and $X_{9}$ (KOOS_Sport), more precisely the internal/external rotation angle at initial contact. The improvement in KOOS_Sport score was identified by Makovey et al. [23] as being related to the changes in the range of motion (ROM) in the transverse plane. Therefore, the model explaining the value of KOOS_Sport and recreation score as a function of the kinematic parameters in the transverse plane and the BMI showed (Table 4) positive correlations.

When comparing the multiple regression models in $\mathbf{C_{1}}$ and $\mathbf{C_{2}}$ (Table 4), we note that they are both related to pain scores ($X_{2}$: Pain Numerical Scale and $X_{7}$: KOOS_Pain) but they are not associated with the same kinematic parameters. Indeed, these two scores, i.e., $X_{2}$ and $X_{7}$, are quite different because they are evaluated based on different symptoms: the Pain numerical Scale variable ($X_{2}$) was evaluated on a 0–10 pain intensity scale and concerns a general pain felt for knees, whereas KOOS_Pain variable ($X_{7}$) was evaluated based on (9) questions, especially relative to the knee injury [10].

## Conclusion

The CCA results showed a moderate correlation that gave rise to three clusters of the most closely related parameters. Multivariate linear models were found complementing the subjective clinical parameters by the biomechanical data using the correlation clusters.

Only the age, BMI, pain which is measured based on Pain Numerical Scale (NS), KOOS_Pain, and KOOS_Sport scores were correlated with the kinematic parameters (mechanical biomarkers). Biomechanical data corresponding to kinematic parameters in the frontal plane (abduction/adduction) during swing phase, the kinematic parameters in the sagittal plane (flexion/extension) at the end of the stance phase, and the kinematic parameters in the transverse plane (internal/external rotation) were positively correlated with pain. In other words, pain increased when kinematic parameters in those planes also increased. On the other hand, Biomechanical data corresponding to kinematic parameters in the frontal plane (abduction/adduction) at initial contact and during the loading phase were correlated negatively with pain. This means a decrease in the frontal plane at those phases is related with an increase in pain level. Kinematic parameters in the transverse plane (internal/external rotation) were correlated positively with the KOOS sport and recreation function. This means that KOOS_Sport increased when movement in the transverse plane was more increased in kinematic parameters.

Finally, the results show that a multivariate analysis of the clinical symptoms and the biomechanical characteristics of knee joint function allows a better understanding of their relationships and would help to better understand how biomechanical characteristics can be used in guiding clinical decision making in OA management.

## Data Availability

The participants data are of the confidential category and cannot be put in an open repository for unrestricted public access. This restriction is imposed by the ethics committee of the University of Montreal Hospital Research Center. However, they can be made available upon request provided a statement of confidentiality is signed. Please contact the Quality system manager Clarisse Bascans (clarisse.bascans@etsmtl.ca).
